# Children with Autism Spectrum Disorder Exhibit Elevated Physical Activity and Reduced Sedentary Behavior

**DOI:** 10.3390/brainsci13111575

**Published:** 2023-11-09

**Authors:** Abdulrahman M. Alhowikan, Nadra E. Elamin, Sarah S. Aldayel, Sara A. AlSiddiqi, Fai S. Alrowais, Wail M. Hassan, Afaf El-Ansary, Farah Ali Alghamdi, Laila Y. AL-Ayadhi

**Affiliations:** 1 Department of Physiology, College of Medicine, King Saud University, P.O. Box 2925, Riyadh 11461, Saudi Arabia; ayadh2@gmail.com; 2 Autism Research and Treatment Center, Department of Physiology, Faculty of Medicine, King Saud University, Riyadh 11461, Saudi Arabia; nadraelyass@hotmail.com; 3 Department of Medicine, College of Medicine, King Saud University, Riyadh 11461, Saudi Arabia; aldayelsarah@gmail.com (S.S.A.); faialrowais@gmail.com (F.S.A.); 4 King Abdullah Bin Abdulaziz University Hospital (KAAUH), Riyadh 11461, Saudi Arabia; sara.alsiddiqi@gmail.com; 5 Department of Biomedical Sciences, University of Missouri-Kansas City School of Medicine, Kansas City, MO 64108, USA; hassanwm@umkc.edu; 6 Autism Center, Lotus Holistic Medical Center, Abu Dhabi P.O. Box 110281, United Arab Emirates; 7 College of Medicine, Dar Al-Olum University (DAU), Riyadh 13314, Saudi Arabia; farahalghamdi1999@gmai.com

**Keywords:** autism spectrum disorder, physical activity, sedentary behavior, ActiGraph monitor, neurochemistry

## Abstract

According to previous research, individuals with autism spectrum disorder (ASD) have lower levels of physical activity than their typically developed (TD) counterparts. There have been conflicting reports about physical activity (PA) levels in people with ASD. Given the conflicting evidence, further investigation is required. We believe that evaluating PA in individuals with ASD is critical in order to offer PA intervention plans aiming at increasing their health-related physical fitness on a daily, systematic, and individualized basis. In the current study, an ActiGraph monitor (GT3X+) was used to accurately measure PA and sedentary activity in 21 children with autism aged 6.43 ± 2.29 years and 30 TD children aged 7.2 ± 3.14 years. Our data indicated that while the light and moderate activity counts were not significantly different between the two groups, the vigorous activity was significantly higher in ASD compared to TD. This finding was attributed to ASD characteristic stereotypy and self-stimulating behaviors. The significantly higher vigorous PA is discussed in relation to altered neurochemistry, oxidative stress, and neuroinflammation as etiological mechanisms in ASD. This research provides a better understanding of the status of PA participation in individuals with ASD.

## 1. Introduction

Autism spectrum disorder (ASD) is a group of neurodevelopmental disorders characterized by social and behavioral deficits, impairment in verbal and nonverbal communication, and restricted, repetitive behaviors and interests [1]. According to the World Health Organization, the global prevalence of ASD in 2022 was 1 per 36 people [2]. The etiology of ASD remains elusive. A combination of multiple etiologies has been suggested, including genetic, epigenetic, immunologic, environmental [3,4], and metabolic abnormalities [5]. Mitochondrial dysfunction, neuroinflammation, and glutamate excitotoxicity are the most strongly implicated etiologies that correlate with the severity of ASD symptoms [6].

A growing body of evidence indicates that children with ASD experience motor function deficits not often seen in their typically developing (TD) counterparts [7]. Motor impairments, including deficits in gross and fine motor skills [8,9], may limit the participation of ASD children in sports and other physical activities (PAs) [10,11,12]. Low levels of physical activity, late motor skills, and fitness, especially in children and adolescents with ASD, may exacerbate social and emotional deficits and related comorbidities [13]. In addition, an association between the severity of motor and social impairments has been reported in children with ASD [14,15,16,17], suggesting that social impairments may also limit ASD children’s participation in PA.

PA, especially moderate and vigorous activities (MVPAs), is important for promoting health and overall quality of life among children, including children with ASD [18,19,20]. Recently, a growing interest in understanding the relationship between social and cognitive features of ASD and physical health outcomes has been widely noted [21]. Previous studies revealed that behavioral functioning in ASD children improved after performing some PA [22,23]. PA has been shown to effectively lower the frequency of stereotypical behavior episodes in children with ASD. It is believed that PA can cause significant changes in brain structure, function, and cognition in ASD children [24]. Long-term physical exercise modifies the structure of motor areas such as the cerebellum and motor cortex, as well as parts of the hippocampus, which is critical for learning, memory, and navigation [25].

Some studies reported that autistic people typically exhibit a reduction in physical activity due to social and behavioral abnormalities. Limited opportunity for exercise impacts the behavior of autistic children, leading to chronic illnesses, such as obesity, which is common in autistic patients [10,26,27]. Furthermore, it has been reported that age and gender may influence the outcome of PA reduction [10,28,29]. Specifically, ASD children’s physiological, cognitive, psychosocial, and behavioral functioning have been found to benefit from moderate to vigorous physical activity (MVPA) [30].

Since the effects of PA on cognition have important implications for improving performance in ASD children, accurate assessment of PA levels is important for studying PA patterns in ASD children. Previous studies have used accelerometer-based activity monitors and self-reported questionnaires to evaluate the level of PA. However, accelerometers have been widely accepted as a more reliable tool for the assessment of a range of PA types in a free-living environment [24,31,32].

One of the most widely used devices in PA research is the ActiGraph monitor (GT3X+), which captures children’s total body movements and free-play activities, including sitting, standing, walking, walking up the stairs, running, and cycling. It can accurately measure the orientation and immature motor movements of a child, making it suitable for the assessment of PA and sedentary activity in persons with disabilities [28,33]. For MVPA, moderate (3–5.99 METs) is defined as 2690–6166 counts/min and vigorous (6 METs) as 6167 counts/min. The total number of minutes of light-intensity physical activity is defined as the total number of minutes between 200 and 2690 counts/min [34].

To provide PA intervention programs aimed at enhancing health-related physical fitness on a daily, systematic, and individualized basis, it is essential to evaluate PA in people with ASD. The current study aimed to determine whether PA has a differential effect on children with ASD compared with their TD age-matched children. Our findings provide novel insights that should inform the development of effective interventional strategies.

## 2. Materials and Methods

### 2.1. Participants

Twenty-one children with autism aged 3–13 years (mean age 6.43 ± 2.29 years) were recruited from the Autism Research and Treatment Center, Faculty of Medicine, King Saud University, Riyadh, Saudi Arabia. A diagnosis of autism was confirmed in all children using the 4th edition of the Diagnostic and Statistical Manual of Mental Disorders. The exclusion criteria were comorbid ASD-related medical diseases or other neurological problems. The exclusion criteria were established by parent interviews and a review of the children’s medical records. The Childhood Autism Rating Scale (CARS), Social Responsiveness Scale (SRS), and sensory profile as measures of severity of the studied participants are presented in Table 1. It can be easily noticed that all are moderate–severe cases. For CARS, scores between 30 and 36.5 indicate mild to moderate autism, and scores from 37 to 60 indicate severe autism [34]. For SRS, 60–75 is considered mild to moderate, and a T-score >75 indicates severe impairment [35].

The control group was comprised of 30 age-matched TD children (mean age 7.2 ± 3.14 years) who attended the pediatric clinic of King Khalid University Hospital, Riyadh, Saudi Arabia, for routine follow-up. They had no clinical signs or symptoms indicative of neuropsychiatric disorders. Children with any neurological, endocrine, cardiovascular, pulmonary, liver, or kidney disease were excluded from the study.

Informed consent for the study was obtained from parents or legal guardians of the investigated subjects as approved by the ethical guidelines of medicine of King Saud University number 13/3945//IRB.

### 2.2. Physical Activity Measurement

Physical activity was measured using the ActiGraph GT3X+ accelerometer (ActiGraph, Pensacola, FL, USA). Parents or caregivers were instructed on how to operate the device and attach it to the child’s waist. Participants were instructed to wear the monitor during all activities except for swimming, showering/bathing, and during their sleep. Parents or guardians were provided with a log to record times when the accelerometer was not worn. Previous studies showed that at least 6 days of recordings were needed for accurate evaluation of sedentary behavior [36,37]. Participants kept the accelerometer for at least 7 consecutive days. To be included in the study, participants had to have accelerometer recordings for a minimum of 6 days, including at least 1 weekend day, and at least 10 h of recordings each day. Participants who did not meet these criteria were excluded from the study. Activity levels were stratified based on accelerometer counts per minute, according to the protocol described by Freedson [38]. Time spent in sedentary behavior, light-intensity physical activity, and moderate–vigorous physical activity (MVPA) was quantified using cut-point thresholds established specifically for preschool children.

### 2.3. Anthropometric Measurements

Participants’ height was measured in centimeters to the nearest tenth of a millimeter. Weight was measured in kilograms to the nearest gram, using an electronic scale (SECA S-214, Basel, Switzerland stadiometer). Measurements were taken twice for each participant, and mean values were recorded. BMI reference values have been set by the World Health Organization (WHO), and their formula was used to assess the quantity of fat in controls and children with autism [39]. The metabolic equivalent of tasks (METs) was estimated using the ActiGraph regression equation developed by Freedson et al. [38], which utilizes counts and age to estimate METs. In the current study, we selected ActiGraph cut points in preschool-age children to estimate metabolic equivalent. To estimate fat percent, the skin folds at the triceps and subscapular area were measured by Lange Skinfold Caliper. Waist and hip circumferences were measured using a measuring tape, and the data were used to calculate the hip-to-waist ratio. Muscle strength was estimated by measuring hand grip strength using Takei Hand Grip Dynamometer. All measurements were taken twice, and averages were recorded.

### 2.4. Statistical Analysis

Data analysis was performed using Statistical Package for Social Studies (SPSS) version 22 (IBM Corp., New York, NY, USA) unless otherwise indicated. Continuous variables were expressed as mean ± standard deviation. Significance of observed differences was evaluated using the *t*-test with normally distributed variables and the Wilcoxon Mann–Whitney test with non-normally distributed variables. Differences associated with a *p*-value < 0.05 were considered significant. Statistical power was estimated using G*Power version 3.1.9.4 [18]. Power was calculated for the study’s sample sizes (21 autism and 30 control) for each of the predictor variables. Correlations were computed using Spearman correlations (r), and a *p*-value was calculated to indicate the significance of the correlation, with a *p*-value < 0.05 indicating significance. When comparing correlation coefficients between groups, multiple computations were performed to determine whether observed differences between group correlation coefficients met statistical significance. First, Fisher z transformation was applied to the pair of correlation coefficients to be compared, r_1_ and r_2_, converting them to the Fisher z scores z_1_ and z_2_, respectively. This was performed using the equation shown below (1). A Z-test was then calculated by dividing the difference between z_1_ and z_2_ by the standard error of that difference, as shown below (2). A *p*-value was calculated, with values < 0.05 indicating statistical significance. Fisher z transformation and the Z-test were performed using the syntax written by Weaver and Wuensch (Weaver 2013) for SPSS.

z_i_ = 1/2 ln ((1 + r)/(1 − r)) (1)

z = (z_1_ − z_2_)/√ (1/(n_1_ − 3) + 1/(n_2_ − 3)) (2)

where z_1_ and z_2_ are Fisher z scores corresponding to correlation coefficients r_1_ and r_2_, and n_1_ and n_2_ are the sample sizes corresponding to r_1_ and r_2_, respectively.

## 3. Results

Demographic and anthropometric data are presented in Table 2. Twenty-one children with ASD and 30 age-matched TD children were included in this study. We found that METs were higher in ASD children compared with TD children (*p* = 0.001). All other demographic and anthropometric characteristics did not significantly differ between the two groups of children.

We observed a modest but statistically significant decrease in total sedentary bouts in ASD children. We did not observe any differences in total activity counts or total time spent (*p* = 0.957) in all types of PA between the two groups.

Our results (Table 3) revealed that there was a highly significant difference in total time spent in sedentary activity (*p* < 0.001) and in the total sedentary activity counts (*p* < 0.001) in the control group compared with the ASD group. The results also indicated that there were no significant differences between groups for the total counts and time spent in LPA and MPA. However, ASD children spent more time than TD engaging in VPA (*p* = 0.017).

**Figure 1:**
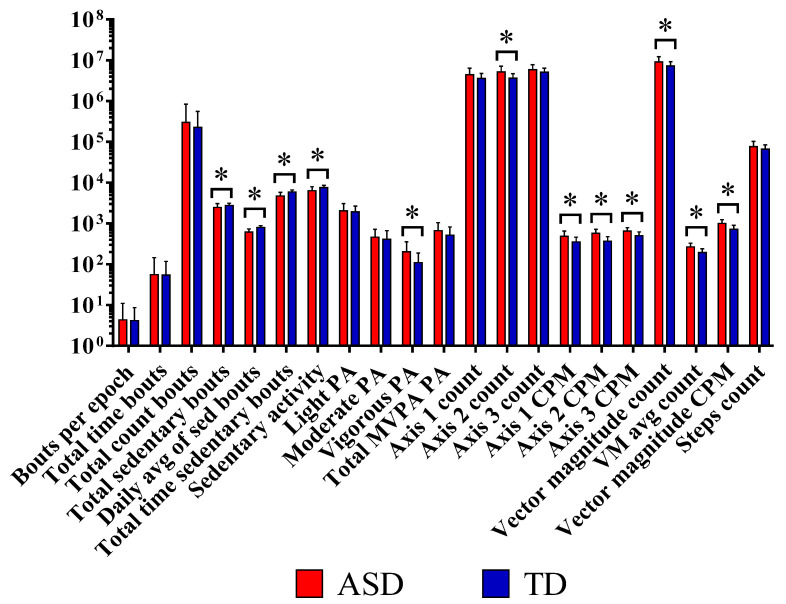
The step counts per day showed higher correlation with vigorous-intensity physical activity (r = 0.759; *p* = 0.001 and MVPA, r = 0.668; *p* = 0.001) than with light- (r = 0.552; *p* = 0.009) or moderate-intensity activity (r = 0.549; *p* = 0.01) among ASD participants. In the control group, the step counts per day were more highly correlated with moderate-intensity physical activity and MVPA (r = 0.633; *p* = 0.001, r = 0.664; *p* = 0.001, respectively) than with vigorous-intensity activity (r = 0.497; *p* = 0.005).

The total counts on different axes showed that PA varied substantially depending on the axis. PA assessed by axis 2 (horizontal or forward and backward motion) showed a highly significant difference in ASD compared to TD (*p* = 0.001), whereas axis 1 (vertical or upward and downward motion) and axis 3 (lateral or left and right motion) showed no significant difference. Total vector magnitude counts (VM counts) were also significantly higher in the ASD group compared with TD (*p* = 0.024). Furthermore, counts per minute (CPMs) were significantly higher in ASD over all axes compared with TD (*p* = 0.001), as well as for the vector magnitude CPM (*p* ≤ 0.001) (Figure 1 and Table 3).

When the correlation between step counts and total energy expenditure in METs was calculated, the correlation was slightly higher in ASD than in the control group (r = 0.768, *p* = 0.001; 0.773, *p* = 0.001). * Significant correlations with *p* < 0.05. 

## 4. Discussion

Research focusing on the association between ASD symptoms and PA has substantially increased over the last few years. Previous research demonstrated that PA has important implications for the improvement of some social, cognitive, and behavioral features [18,19,20,21]. To the best of our knowledge, the current study is the first to assess sedentary behavior and physical activity in Saudi ASD children.

The precise assessment of physical activity levels is crucial for understanding the association between active lifestyle and health, especially when evaluating the effectiveness of intervention programs [13,22]. By assessing the level of PA for autistic children, it is possible to develop sports programs that support the health of this group. In the current study, the overall time spent in PA and the total activity level did not differ significantly between ASD and controls. This result agrees with previous studies. Sandt and Frey reported no differences between ASD children and controls in any physical activity setting [40].

### 4.1. Sedentary Activity in ASD Participants

In this study, ASD children spent less time in sedentary activity (SA) than TD (*p* = 0.001). This finding is in line with some previous studies comparing children with different disabilities, including ASD, with TD children in Europe and North America. For example, it has been reported that young children with ASD are more active and spend significantly less time in sedentary behavior compared with the control group [26,41], suggesting that the PA differential between ASD and TD may be age-related [11,14,28]. In contrast, other studies reported that ASD children were less physically active and had an increased sedentary lifestyle compared with TD, as sedentary activity ASD children may experience impairments in movement, communication, social skills, and behavior [10,11].

Our data indicated that the light and moderate activity counts were not significantly different between the two groups, but vigorous activity was significantly higher in ASD compared with TD. This finding contrasts with previous studies [11,42,43]. Researchers demonstrated that ASD children spent less time in PA compared with TD children. This finding could be attributed to their characteristic stereotypical and self-stimulating behaviors. They concentrate on negative habits, which prevent them from engaging in physical activity. It was noticed that the time spent in MVPA did not differ significantly between the studied groups (*p* = 0.132). It only accounts for a small amount of time in the day, and the majority of the time was spent in LPA and sedentary behavior.

Table 4 shows a significantly stronger correlation between daily step counts and all physical activity intensities (time spent in light, moderate, and vigorous activity) in the ASD group than in the control group, despite the fact that there was no statistically significant difference between the two groups in terms of step counts. This result shows that the group with higher step counts spent a lot more time being physically active.

### 4.2. Vigorous PA in ASD Patients

It would be intriguing to investigate the association between increased vigorous PA in ASD patients compared with TD children and unbalanced excitatory/inhibitory neurotransmission, oxidative stress, and neuroinflammation as ASD etiological processes related to hyperactivity [44,45]. Individuals with autism have substantially greater glutamate, the primary excitatory neurotransmitter, and much lower gamma amino butyric acid (GABA), the primary inhibitory neurotransmitter [6,46]. Under normal physiological conditions, released glutamate is metabolized or taken up by neighboring astrocyte cells through glutamate transporters. When these pathways are disturbed, as in ASD, glutamate builds up and overexcites the N-methyl-D-aspartate (NMDA) receptors. These receptors, when triggered by excessive glutamate, function as a Ca^2+^ (calcium ion) channel. Because Mg^2+^ (magnesium) blocks the channel, these channels only operate when the cell membrane is depolarized. In ASD, the membrane is chronically depolarized, Mg^2+^ exits the channel, and Ca^2+^ influx is unrestricted for longer periods of time, leading to cell death via free radicals [47] or through mitochondrial overload, which results in free radical formation. Inflammatory mediators, increased oxidative stress, and decreased levels of brain-derived neurotrophic factor (BDNF) and other growth factors have more or less similar effects on glutamate microcircuits in ASD patients, regardless of whether the origin is centrally or peripherally derived. When mitochondria become damaged and electron leakage increases, ROS generation increases [48]. Theoretically, the higher PA in ASD patients could be due to the idea that the brain can use elevated glutamate as an energy source to dispose of the neurotransmitter’s excess levels. This explanation can find support by considering the fact that NMDA receptor antagonists reduce channel permeability and inhibit Ca^2+^ influx, providing neuroprotection, amending glutamate excitotoxicity, and perhaps ameliorating hyperactivity symptoms [44,49].

### 4.3. Vigorous PA and Sleep Disruptions in ASD Patients

Sleep disruptions are one of the most common comorbidities reported in ASD children, occurring in up to 80% of ASD children compared with 20–40% of typically developing children [50,51,52], and include insomnia, circadian rhythm disturbances, difficulty falling asleep, restless sleep, and frequent waking [53]. Most sleep comparison studies using objective (actigraphy) or subjective (questionnaires) assessments have found that children with ASD had lower sleep metrics than their typically developing counterparts [54]. In an attempt to find a link between the recorded increased aggressive PA and sleep problems in autistic individuals, it was interesting to consider the work of Wang et al. [54], who reported that some children with much higher PA have impaired sleep latency, bedtime resistance, and awakening latency. It was documented that minimal or excessive PA had a negative impact on sleep quality and quantity. Although sedentary living has frequently been suggested to explain this sleep disruption, it has been less frequently proven that an excess of PA may be deleterious. This can support the harmful effect of the increase in aggressive PA in our ASD participants compared with controls [55]. The suggested link between increased aggressive PA, glutamate excitotoxicity as a neurochemical characteristic of an autistic brain, and sleep disruption as a comorbidity in ASD patients could find support in the work of Bell et al. [56], in which they proved the relationship between glutamate excitotoxicity and sleep deficits in EcoHIV-infected mice.

### 4.4. Limitations

Our well-defined sample of children with a thorough diagnosis of ASD is one of our work’s strengths. However, our study has a number of limitations. First, it is descriptive; it does not investigate the impact of PA on selected comorbidities of ASD, such as anxiety, stress, and sleeping difficulties. Second is the small sample size, and our population was limited to ASD, which restricts the generalizability of the current findings. However, given the scarcity of studies in this field, our poor understanding of this group, the possibility for objective measurement practices, and potential correlations between PA and quality of life, the findings must be reported, replicated, and expanded.

## 5. Conclusions

The major finding of this study was that the ASD children were more physically active and less sedentary than TD. The outcomes of the current study should be interpreted with caution due to the small sample size. Therefore, larger studies with larger samples are needed to explore the potential role of physical activity engagement in the improvement of cognition and brain function in ASD children.

## Figures and Tables

**Table 1:** CARS and SRS as measures of severity of the twenty-one children with ASD recruited in the study.

Patient ID	CARS	SRS
31	37	98
32	36	99
33	38	61
34	36	66
35	34	66
36	35	65
37	40	77
38	39	80
39	38	36
40	36	44
41	35	47
42	36	56
43	38	55
44	40	35
45	41	33
46	37	79
47	35	77
48	35	43
49	36	44
50	34	46
51	34	89

**Table 2:** Demographic and anthropometric characteristics of autism and control groups (mean ± SD). * *p* < 0.05.

Variable	Autism (N = 21)	Controls (N = 30)	*p*-Value	Power
Age (years)	6.43 ± 2.29	7.20 ± 3.14	0.342	0.59
Weight (kg)	27.24 ± 11.45	27.81 ± 11.04	0.861	0.50
Height (cm)	125.19 ± 15.73	126.96 ± 16.28	0.706	0.48
BMI (kg/m^2^)	16.75 ± 3.68	16.60 ± 2.37	0.870	0.50
Percentile	48.91 ± 39.60	51.48 ± 31.64	0.804	0.51
Waist (cm)	57.20 ± 11.17	58.26 ± 9.40	0.726	0.51
Hip (cm)	68.40 ± 10.59	69.78 ± 9.72	0.646	0.52
Waist-to-hip ratio	0.83 ± 0.08	0.83 ± 0.04	0.981	0.50
Skin fold—triceps (mm)	12.01 ± 7.97	10.32 ± 5.24	0.396	0.58
Skin fold—subscapular (mm)	11.04 ± 8.06	7.44 ± 4.24	0.059	0.77
Hand grip strength KG	6.59 ± 0.82	9.09 ± 3.71	0.069	0.92
METs	1.99 ± 0.32	1.63 ± 0.22	0.001 *	0.98

**Table 3:** Means and SD of ActiGraph variables in autism and control groups. * *p* < 0.05.

Variable	Autism (N = 21)	Controls (N = 30)	*p*-Value	Power
Bouts per epoch	4.33 ±6.71	4.13 ± 4.54	0.899	0.50
Total time bouts	55.49 ± 90.23	54.32 ± 63.42	0.957	0.50
Total counts bouts	298,698.14 ± 536,651.88	226,350.43 ± 329,120.57	0.554	0.53
Total sedentary bouts	2476.95 ± 598.25	2752.23 ± 365.64	0.047 *	0.77
Daily avg. sed. bouts	615.53 ± 121.37	790.63 ± 90.16	<0.001 *	1.00
Total time sedentary bouts	4703.20 ± 1099.91	5934.34 ± 702.18	<0.001 *	0.98
Sedentary activity	6307.04 ± 1622.23	7570.66 ± 1003.51	0.001 *	0.93
Light PA	2039.48 ± 1014.75	1925.73 ± 754.36	0.648	0.52
Moderate PA	462.85 ± 257.34	406.98 ± 257.54	0.449	0.56
Vigorous PA	201.55 ± 152.70	109.23 ± 79.08	0.017 *	0.87
Total MVPA PA	664.39 ± 384.90	516.20 ± 304.78	0.132	0.69
Axis 1 counts	4,435,262.71 ± 1,901,170.01	3,545,304 ± 1,192,223	0.067	0.77
Axis 2 counts	5,192,411.81 ± 1,866,204.11	3,646,306 ± 1,029,070	0.001 *	0.95
Axis 3 counts	5,873,091.76 ± 1,898,634.97	5,033,960 ± 1,362,121	0.092	0.74
Axis 1 CPM	484.20 ± 166.20	352.66 ± 107.74	0.007 *	0.93
Axis 2 CPM	570.22 ± 144.02	366.19 ± 104.44	<0.001 *	1.00
Axis 3 CPM	645.21 ± 140.03	501.40 ± 120.83	<0.001 *	1.00
Vector magnitude counts	9,040,543 ± 3,172,723	7,184,405 ± 1,977,732	0.024 *	0.85
VM avg. counts	264.72 ± 65.30	191.96 ± 47.96	<0.001 *	0.98
Vector magnitude CPM	991.62 ± 245.30	716.92 ± 181.32	<0.001 *	0.98
Steps counts	75,742 ± 27,182	65,762 ± 18,197	0.152	0.69

**Table 4:** Pearson correlation coefficient (r) of step counts per day with the time spent in physical activity in ASD and TD groups, and with METs.

Variable	ASD		TD		Statistical Significance of the Differences between ra and rt	
	ra	*p*-Value	rt	*p*-Value	Test Statistic (z)	*p*-Value
Time spent in light-intensity activity	0.552	0.009	0.040	0.8	n/a	n/a
Time spent in moderate-intensity activity	0.549	0.01	0.633	0.001	−0.4254	0.6705
Time spent in vigorous-intensity activity	0.759	0.001	0.497	0.005	1.4740	0.1405
Time spent in MVPA	0.668	0.001	0.664	0.001	0.0236	0.9812
METs	0.768	0.001	0.773	0.001	−0.0404	0.9677

## Data Availability

The dataset supporting the conclusions of this article is available upon request to the corresponding author. Email: ahowikan@ksu.edu.sa.
